# Proprioceptive Neuromuscular Facilitation and/or Electrical Stimulation in Patients with Peripheral Facial Paralysis: A Systematic Review

**DOI:** 10.3390/neurolint17020017

**Published:** 2025-01-23

**Authors:** Nerea Dominguez-Defez, Juan Lopez-Barreiro, Pablo Hernandez-Lucas, Ana González-Castro

**Affiliations:** 1 Nursing and Physical Therapy Department, University of Leon, Astorga Ave., 24401 Ponferrada, Spain; ndomid00@estudiantes.unileon.es (N.D.-D.); agonzc28@estudiantes.unileon.es (A.G.-C.); 2 Faculty of Education and Sport Sciences, University of Vigo, Campus A Xunqueira, 36005 Pontevedra, Spain; juan.lopez.barreiro@uvigo.es; 3 Research Group HI10, Department of Functional Biology and Health Sciences, Faculty of Physiotherapy, University of Vigo, Campus A Xunqueira, 36005 Pontevedra, Spain

**Keywords:** Bell’s palsy, facial nerve diseases, rehabilitation, exercise therapy, musculoskeletal manipulations, electrotherapy

## Abstract

Background: Peripheral facial paralysis (PFP) affects the facial nerve, the seventh cranial nerve. It has an incidence rate of 20–30 cases per 100,000 habitants. The diagnosis is clinical, though imaging tests may be required in some cases. The treatment protocol includes medication, physiotherapy, and, in certain cases, surgery. Proprioceptive neuromuscular facilitation (PNF) techniques and electrical stimulation have been shown to be significant for recovery. Although PFP has a high recovery rate, up to 40% of patients may experience permanent sequelae. Objective: to assess the efficacy of treatment based on electrical stimulation and/or PNF in patients affected by PFP. Methods: A systematic search was conducted across six databases (PubMed, Medline, SportDiscus, CINAHL, Scopus, and Web of Science) in November 2024. Randomized controlled trials were included. Results: Fourteen articles were analyzed, applying PNF and/or electrical stimulation methods, pharmacological treatment, low-level laser treatment, subcutaneous collagen injections, and physiotherapy protocols involving facial expression exercises, yielding evidence for the variables assessed. Conclusions: PNF and/or electrical stimulation treatment in patients with PFP can be effective when employed early with appropriate parameters, showing promising results in improving quality of life, facial movement quality, and CMAP and reducing both the incidence and degree of synkinesis.

## 1. Introduction

Facial paralysis is characterized by a reduction in or absence of the ability to move the facial muscles due to impairment of the facial nerve, also known as the seventh cranial nerve. Among its types, peripheral facial paralysis (PFP) is the most common and arises from the denervation of the nerve trunk along its course from the brainstem to its terminal branches in the face [1,2]. The incidence of PFP is estimated at 20–30 cases per 100,000 individuals, representing approximately 40,000 new cases annually worldwide [3]. It affects both men and women equally and can present on either side of the face, although it is more frequently observed in pregnant women or those of reproductive age due to hormonal factors [3,4,5,6].

The diagnosis of PFP is primarily clinical, based on a detailed medical history that includes the patient’s background and symptoms [7]. In addition to evaluating facial movements, the physical examination includes an inspection of the oral cavity, a visual assessment of the tympanic membrane, and palpation of the carotid artery, as well as checking for lymphadenopathy [4,8]. A neurological examination is also recommended to rule out central lesions and identify potential abnormalities in other cranial nerves [9]. In atypical cases or when the diagnosis is uncertain, imaging studies such as computed tomography or magnetic resonance imaging may be used to exclude other pathologies [8].

The management of PFP encompasses both conservative and surgical treatments. Conservative options include medications aimed at improving blood circulation, controlling infections, and promoting nerve regeneration. These may include antibiotics, anti-inflammatory drugs, and vitamins. In selected cases where nerve entrapment or compression is present, surgical decompression via a middle cranial fossa approach may be employed and has shown efficacy [10].

Physical therapy plays a crucial role in PFP recovery. Common therapies include facial massages and exercises designed to enhance muscle tone, improve blood flow, and restore muscle function to reduce facial asymmetry [9]. However, even with appropriate management, it is estimated that 40% of patients may develop sequelae such as muscle weakness, synkinesis, chronic pain, and asymmetries, significantly impacting their quality of life and emotional and social well-being [11].

In this context, more recent techniques, such as electrical stimulation and proprioceptive neuromuscular facilitation (PNF), have emerged as promising options. Electrical stimulation aids in improving blood circulation, increasing muscle strength and endurance, and optimizing muscle fiber recruitment for more effective contractions [12]. On the other hand, PNF involves global stretching, verbal commands, resistance, and manual contact to facilitate muscle contraction in the affected muscles [13]. This approach relies on the activation of stored motor patterns, eliciting a more efficient muscle response. Additionally, the application of manual resistance to stronger muscles induces energy irradiation towards weaker muscles, enhancing their activation [14].

In light of these advancements, a systematic review was proposed to evaluate the effectiveness of treatments based on electrical stimulation and/or PNF in patients with PFP.

## 2. Materials and Methods

### 2.1. Protocol and Registry

The systematic review was conducted following the PRISMA (Preferred Reporting Items for Systematic Reviews and Meta-Analyses) guidelines and the PERSIST (Prisma Implementation in Exercise, Rehabilitation, Sports Medicine, and Sports Science Topics) recommendations [15,16]. It was prospectively registered in PROSPERO (CODE: CRD42024611595). The included studies addressed the PICOS question: population (patients with PFP or Bell’s palsy), intervention (PNF and/or any form of electrical stimulation), comparison (pharmacological treatment and/or other physical therapy techniques or methods), outcomes (amount and/or degree of facial synkinesis and/or health-related quality of life), and study design (randomized controlled trials).

The following inclusion criteria were applied: (a) samples exclusively consisting of patients diagnosed with PFP; (b) interventions under evaluation that included PNF and/or electrical stimulation; (c) control groups treated with some form of medication and/or active exercise therapy; (d) study variables that included facial synkinesis and/or health-related quality of life; (e) studies consisting of randomized controlled trials. No exclusion criteria were defined. No additional exclusion criteria were defined, as studies that did not meet the inclusion criteria mentioned were excluded during the selection process.

### 2.2. Source of Information

The databases consulted in November 2024 were PubMed, Medline, SportDiscus, CINAHL, Scopus, and Web of Science. The search strategy was designed using terms derived from the Medical Subject Headings (MeSH) thesaurus: Bell’s palsy, facial nerve diseases, cranial nerve, cranial nerve diseases, facial paralysis, facial nerve, facial nerve injuries, rehabilitation, myofunctional therapy, physical therapy modalities, electric stimulation therapy, exercise therapy, musculoskeletal manipulations, and exercise movement techniques.

These terms were combined with free-text terms: facial palsy, facial nerve palsy, peripheral facial paralysis, exercise, myofunctional exercises, Kabat, electrotherapy, and manual therapy. All of them were combined using Boolean operators “OR” or “AND” (Supplementary Material S1).

### 2.3. Study Selection

The selection process began with an initial screening of all titles and abstracts of the identified results, applying the pre-defined inclusion criteria. Two authors independently participated in this stage. In the event of discrepancies between them, they were resolved through discussion until a consensus was reached.

Subsequently, the full texts of the preselected publications were analyzed, with the inclusion criteria reapplied to confirm their relevance. Additionally, during the database search, systematic reviews were identified, and their reference lists were examined to locate additional primary studies. These studies were also assessed according to the inclusion criteria, first through their titles and abstracts and then in their full texts.

### 2.4. Data Selection Process

The data extraction from the selected studies was conducted independently by two authors, who collected key information regarding aspects such as authorship and publication year, the study’s objectives and methodology, sample characteristics (including size, sex distribution, and inclusion/exclusion criteria), and details of the intervention (applied techniques and methods, duration, number and frequency of sessions), as well as assessments performed, instruments used, evaluation times, and results obtained. In cases of discrepancies during this process, both authors engaged in discussions until a mutual agreement was reached, thus ensuring the accuracy and consistency of the collected data.

### 2.5. Methodological Quality Assessment

The methodological quality assessment of the studies was performed using the Physiotherapy Evidence Database Scale (PEDro) [17], and the risk of bias was determined using the Cochrane risk of bias tool for randomized trials (RoB 2) [18]. This process was independently conducted by two authors. In cases of discrepancies in scoring or interpretation, they resolved them through discussion until a consensus was reached.

## 3. Results

### 3.1. Identified Studies

**Figure 1:**
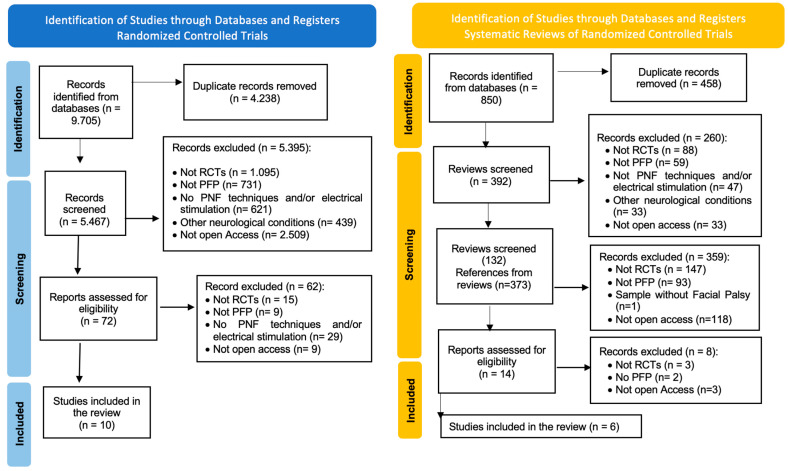
Preferred Reporting Items for Systematic Reviews and Meta-Analyses (PRISMA) flow diagram. RCT: randomized controlled trial.

Following the search strategies, a total of 9705 results were identified. Of these, 4238 were duplicates. After the initial screening of titles and abstracts from the remaining 5467 articles, 72 articles were selected. Following a full-text analysis, a total of 10 articles were obtained. Concurrently, a total of 850 systematic reviews were identified, with 458 being duplicates. After verifying the references of 392 reviews, 132 reviews with 373 references were obtained. Following the initial title and abstract screening, 14 references were selected. After repeating the same full-text screening processes, six new references were selected (Figure 1).

The results obtained using the PEDro scale [17] indicated that two articles received scores below six, while 14 articles achieved scores equal to or greater than six points (Table 1). The RoB 2 scale [18] revealed that 13 articles had a high risk of bias (81.3%), and three articles presented a low risk of bias (18.8%) (Table 2).

Of the interventions evaluated, seven included electrotherapy [19,21,22,27,29,31,33], five employed PNF techniques [20,23,24,28,34], and four used a combination of both [25,26,30,32]. Additionally, 10 studies combined these interventions with other physical therapy techniques and/or pharmacological treatments [19,21,22,23,24,25,26,28,33,34] (Table 3).

### 3.2. Participant Characteristics

Overall, the studies recruited a total of 726 participants, comprising 252 women and 259 men. The sex of the remaining 215 participants was not specified [22,24,27,29,31,32]. This total includes participants from both randomized controlled trials and systematic reviews. One study exclusively included children aged between 11 and 14 years [32], while another only selected individuals over 65 years old [24].

The duration of PFP was chronic in three cases [25,27,28], whereas it was acute in the remaining 12 cases [19,20,21,22,23,24,26,29,30,31,32,33]. The severity of dysfunction reported in 11 studies ranged from moderate to severe [19,20,22,23,24,26,27,28,30,32,33], while in two studies, only severe cases were included [21,25].

### 3.3. PNF Interventions

Of the five articles that applied PNF without combining it with electrical stimulation [20,23,24,27,34], three used it to facilitate voluntary response of the affected muscles through a global pattern in a muscle section exhibiting resistance [20,23,28]. Three regional points were considered: superior, intermediate, and inferior. The superior region included the forehead and eyes connected by a vertical axis; the inferior region encompassed mimic, masticatory, and articulatory muscles along the horizontal axis; and the intermediate region (nose) participated in movements involving both. Manipulation of fulcrums was performed using contralateral contractions, proprioceptive stimulation, stretching, maximum resistance, manual contact, and/or verbal instruction.

With the superior fulcrum, the frontal, corrugator, and orbicular muscles were activated by traction in either a cranial or a caudal direction (depending on the desired action). The intermediate fulcrum activated the levator labii superioris and nasalis muscles through opposing traction. The inferior fulcrum activated the risorius and orbicularis oris muscles in a more horizontal plane, while the mentalis muscle was engaged in a vertical plane [20,23,28]. Monini et al. [24] did not describe the specific techniques used.

Other studies combined PNF with pharmacological treatments [20,23,24,28,34], tailored to each study: antivirals (acyclovir: 400 mg three times daily) and steroids (prednisone: 40 mg/day) for 10 days, gradually reducing the dose from day five onward [20]; steroids (60 mg/day), eye drops, and paracetamol [23]; 60 mg/day of steroids for 10 days (with a 10 mg daily reduction until discontinuation) [24]; or subcutaneous collagen injection (porcine origin) [28]. This last method is the only one that fully detailed its application technique, utilizing an insulin-type syringe with a 30-gauge needle, injecting 0.4 cm^3^ per point of solution into the orbicularis oculi (1 cm lateral to the orbital rim) and the orbicularis oris (at the boundary between the peripheral and marginal areas, approximately 5 mm above the upper and lower vermilion borders) [28].

Additionally, in the study by Özden et al. [34], pharmacological treatment was combined with PNF through telerehabilitation.

### 3.4. Electrical Stimulation Interventions

Seven interventions applied different types of electrical stimulation [19,21,22,27,29,31,33]. These included continuous subthreshold fixed biphasic pulse stimulation [22], low-frequency electrical stimulation combined with simultaneous shortwave [27], transcutaneous electrical nerve stimulation (TENS) [33], and low-level laser therapy [29,31], and in two studies, the specific type of stimulation was not disclosed [19,21]. Notably, Cai et al. [19] did not provide any specific details regarding the intervention. Pulse duration varied across the studies, ranging from 100 ms, a minimum of 10 ms, and a maximum of 50 ms to 700 µs and 10 µs. The applied frequencies included 10 Hz, 20 Hz, and 80 Hz with fixed pulses and between 1 Hz and 3 Hz. Only two of the five interventions reported the intensity [22,27]. Marotta et al. [27] started at 0.5 mA until visible muscle contraction was achieved, while the other study indicated values of 20 mV and 10 V [22].

Regarding electrode placement, Tuncay et al. [21] used rubber electrodes, positioning the anode on each muscle and the cathode on the proximal part of the ipsilateral arm. They stimulated 11 facial muscles, including the corrugator, superciliary, palpebral portion of the orbicularis oculi and oris, levator anguli oris, superioris, risorius, depressor anguli oris, inferioris, and frontalis. Kim and Choi [22] used surface electrodes (2 cm in diameter), placing the cathode on the main branches of the facial nerve and the anode on the mastoid process. Marotta et al. [27] specified placement over the orbicularis oris, frontalis, and zygomaticus muscles, whereas Tuncay et al. [21] did not specify electrode positions.

Electrical stimulation interventions were combined with pharmacological treatment [19,22], conventional physical therapy [21], facial exercises and massage [19,21,27,29,31,33], and facial nerve mobilization techniques [33].

Pharmacological treatments varied between the two studies. One administered neurotrophic agents and vitamins B_12_ and B_1_, while the other used steroids for five days with gradually reduced doses and antiviral treatment (acyclovir) for another five days [22].

Tuncay et al. [21] employed conventional physical therapy, including compensation strategies, posture adjustments, diet modifications, and warm compress applications.

Of the interventions that included facial exercises and massage [19,21,27,33], Barbara et al. [20] employed self-applied massage, conducted two to four times daily for five minutes per session, and a specific training regimen for four regions, forehead, periorbital, nasal, and perioral areas, for two weeks. Tuncay et al. [21] instructed patients on proper facial expression exercises in front of a mirror, performed five times a week for three weeks, starting with balloon blowing and chewing gum on the paralyzed side, accompanied by facial massages. Marotta et al. [27] included mimetic therapy exercises, which involved different breathing, phonation, relaxation, and eye and lip opening and closing exercises, though the specifics were not detailed, combined with facial massage. Alharbi et al. [33] incorporated 10 min massages on both sides of the face and neck and prescribed 10 facial expression exercises to be performed at home three to four times daily, each for at least 10 min.

Additionally, Alharbi et al. [33] implemented facial nerve mobilization techniques, positioning the patient in a supine position with the physiotherapist at the head. One hand facilitated mobilization, while the other stabilized the unaffected side of the head. Mobilization involved circular movements behind the ear, with the thumb positioned on the external auditory meatus. The movement was circular with horizontal traction, applied 25 times in three sets, with rest periods ranging from 5 to 30 s between sets.

### 3.5. Combined Interventions of Electrotherapy and PNF

In four articles [25,26,30,32], a combination of electrotherapy and PNF was employed.

Hamed et al. [32] implemented a PNF program that included the regional fulcrums: superior, intermediate, and inferior, following the same procedure described by Barbara et al. [20], Monini et al. [23], and Micarelli et al. [28]. Additionally, Kabat motor control was applied, lasting 20 min per session, with visual cues in front of a mirror, verbal prompts, and tactile cues through manual therapist contact to guide and teach muscle contraction on the affected side. For electrical stimulation, a faradic current at 100 Hz frequency was used, with 10 s of ramp-up and three seconds of ramp-down; the pulse time was 100 μs, and the pause time was 1 ms, with an intensity that produced visible muscle contractions for two minutes per point, totaling 15 min per session. The positive electrode was placed on the nerve trunk and the negative electrode on the motor points of the frontal, orbicularis oculi, orbicularis oris, nasalis, zygomaticus major, and mentalis muscles. Facial expression exercises were also included, such as gently and tightly opening and closing the eyes, raising the eyebrows, smiling, snarling, flaring the nostrils, pursing the lips, and pouting, with 10 repetitions per exercise, performed for 15 min, twice a day.

The other studies also applied combined PNF therapy with electrical muscle stimulation [25,26,30] but did not specify the parameters used. Additionally, two of these studies included conventional early rehabilitation and exercises [25,30], though they also did not elaborate on the parameters employed.

### 3.6. Control Interventions

Out of the 16 studies, nine utilized pharmacological treatment [19,20,21,22,23,24,25,33,34], seven included active exercise [21,25,26,27,28,30,32], five applied electrical stimulation [19,26,30,32,33], three incorporated massage [21,27,33], one used PNF [28], two applied low-level laser therapy [29,31], and one employed a neuromuscular taping technique [25].

In the studies that used pharmacological treatment [19,20,21,22,23,24,25,33,34], corticosteroids were applied in three [21,25,33]. In the remaining studies, the pharmacological treatment was the same as that used in the experimental groups [19,20,22,23,24,34].

Regarding the studies that included exercise in the control group [21,25,26,27,28,30,32], Tuncay et al. [21] and Marotta et al. [27] implemented a program similar to the experimental group. Similarly, Hamed et al. [32] conducted a facial expression exercise program identical to that of the experimental group, though in this case, it was performed for 35 min. The remaining studies did not detail the exercise programs used [25,26,28,30].

Electrical stimulation, applied in five studies [19,26,30,32,33], was not described in three [19,26,33]. The other two used the same parameters as in the experimental group [30,32].

Of the three studies that involved massage, none provided details on the specific techniques used [21,27,33]. The study that applied PNF did so using three fulcrums [28].

Ghous et al. [25] employed neuromuscular taping with kinesio-taping but did not describe the technique used.

### 3.7. Results on the Quantity and/or Degree of Fascial Synkinesis

For the assessment of the quantity and/or degree of synkinesis, the Sunnybrook Facial Grading System (SFGS) was used to evaluate voluntary movement, symmetry at rest, and synkinesis [22,26,27,32,33]. Two questionnaires were also utilized: the Synkinesis Assessment Questionnaire (SAQ) to assess synkinesis in different muscle groups [25], and the Beta FaCE Scale (BFS), which measured the severity of paralysis and recovery of facial function over time [24].

In seven studies [22,24,25,26,27,32,33], a reduction in the degree of synkinesis was observed at the end of the intervention, although the results were not elaborated upon. Of these, six showed a significant reduction [24,25,26,27,32,33].

A significant difference was noted in the subgroup of participants over 65 years old in two domains: facial movement and oral function. The younger group, on the other hand, showed increased scores in all six domains (facial movement, facial comfort, oral function, ocular comfort, tear control, and social function) of the BFS [24].

The SFGS showed an increase in scores at the end of treatment for voluntary movement symmetry [26,27] and resting symmetry [26]. Another study reported a significant increase at the end of the treatment but did not specify which domains [32]. One study indicated that mean scores changed after three weeks of treatment [33].

The SAQ showed significant post-intervention results, with reduced scores [25].

In the control group, two studies reported significant reductions in scores at the end of the sessions [26,32]. The remaining five studies did not show significant differences [22,24,25,27,33].

In intergroup comparisons, Khanzada et al. [26] found significant differences in both groups, with better outcomes in the domains of symmetry and voluntary movement in the SFGS when PNF combined with electrical stimulation was applied compared to facial exercises combined with electrical stimulation. Hamed et al. [32] reported a total score of 86 in the three subscales at the end of the treatment, compared to 70.66 in the control group.

### 3.8. Results for Movement Quality and Facial Synkinesis

For the assessment of the quantity and/or degree of synkinesis, the SFGS was used to evaluate voluntary movement, symmetry at rest, and synkinesis [22,26,27,29,31,32,33]. Two questionnaires were also utilized: the SAQ to assess synkinesis in different muscle groups [25] and the BFS, which measured the severity of paralysis and recovery of facial function over time [24].

In nine studies [22,24,25,26,27,29,31,32,33], a reduction in the degree of synkinesis was observed at the end of the intervention, although the results were not elaborated upon. Of these, six showed a significant reduction [24,25,26,27,32,33]. A significant difference was noted in the subgroup of participants over 65 years old in two domains: facial movement and oral function. The younger group, on the other hand, showed increased scores in all six domains (facial movement, facial comfort, oral function, ocular comfort, tear control, and social function) of the BFS [24].

The SFGS showed an increase in scores at the end of treatment for voluntary movement symmetry [26,27] and resting symmetry [26]. Another study reported a significant increase at the end of the treatment but did not specify which domains [32]. One study indicated that mean scores changed after three weeks of treatment [33].

The SAQ showed significant post-intervention results, with reduced scores [25].

In the control group, two studies reported significant reductions in scores at the end of the sessions [26,32]. The remaining five studies did not show significant differences [22,24,25,27,33].

In intergroup comparisons, Khanzada et al. [26] found significant differences in both groups, with better outcomes in the domains of symmetry and voluntary movement in the SFGS when PNF combined with electrical stimulation was applied compared to facial exercises combined with electrical stimulation. Hamed et al. [32] reported a total score of 86 in the three subscales at the end of the treatment, compared to 70.66 in the control group.

The House–Brackmann (H-B) scale was used to globally assess the quality of movements and synkinesis [19,20,21,22,23,24,25,28,34]. Eight studies identified a reduction in synkinesis and an improvement in movement quality [19,20,21,22,23,24,25,28]. Seven of these studies reported significant improvements [20,21,22,23,24,25,28,34].

Statistically significant clinical staging differences were observed on days 4, 7, and 15. During this period, seven patients initially presented with an H-B grade IV, and one patient presented with an H-B grade V. By the end of the treatment, overall improvement was noted, with one patient improving from H-B grade V to grade III, three patients improving from grade IV to grade III, and four patients improving from grade IV to grade II [20]. In another study, a reduction from H-B grade III to H-B grade I was observed at the end of treatment [21]. One study reported a four-grade reduction in 25% of participants [23]. Another investigation showed significant differences starting two weeks after the onset of paralysis, persisting for six months. In another study, a four-grade decrease in H-B was noted, with 50% of participants aged over 65 achieving H-B grade I, while the other 50% improved by two grades to H-B grade III. For the younger subgroup, 58.3% achieved H-B grade II with a three-grade reduction, and 41.7% reached H-B grade I with a four-grade reduction by the end of treatment [24].

Two studies did not elaborate on their results [25,28]. Among those without significant differences, one study revealed that the minimum follow-up period for complete recovery was one year, where 100% of participants with mild injury recovered, 94.6% of those with moderate injury recovered, and 31.1% of those with severe injury recovered [19].

In all eight studies [19,20,21,22,23,24,25,28], the control groups showed a reduction in synkinesis. However, none of these control cases demonstrated a significant difference.

### 3.9. Results on Quality of Life

Quality of life was assessed in nine studies [19,21,22,25,26,28,29,30,31]. The scales used included the Facial Disability Index (FDI) [19,21,22,25,26,28,29,30,31,34] and the Quantitative Facial Function Estimation System, as well as the Italian version of the SF-36 [28].

Within the experimental group, seven studies [21,25,26,28,29,30,31] reported significant improvements in FDI results, and one study reported significant improvements in SF-36 scores [28]. Two studies [19,22] did not show significant results.

At the end of the interventions, the social and physical well-being domains of the FDI scale showed significant improvement in four studies [21,26,32,34]. There was an increase in physical function scores, reflecting better voluntary muscle movement [21,26,32] and social function [21,26]. Four studies showed an increase in FDI scores without detailing the specific domains [25,28,29,31].

The SF-36 scale indicated improvements across six items: physical function, social function, mental health, health change, vitality, and general perception [28].

In the control group, five studies showed significant improvement [21,26,28,29,30], while the remaining four did not report any results in this area [19,22,25,31]. Four of these studies highlighted significant differences in the FDI scale’s social and physical well-being domains [21,26,28,30]. The SF-36 indicated a significant improvement in physical and role limitations [28].

When comparing groups, Tuncay et al. [21] demonstrated higher physical state outcomes in the experimental group compared to the control group. Khanzada et al. [26] found that the Kabat technique was clinically superior to muscle retraining for improving social and physical domains. Micarelli et al. [28] showed that both groups improved significantly, but better final treatment outcomes were observed with the combined application of PNF and collagen injection compared to PNF alone, with a shorter recovery time noted. Avaid et al. [30] demonstrated that combined electrical stimulation and PNF techniques improved social and physical function compared to conventional physical therapy.

### 3.10. Other Analysed Variables

The disability resulting from disorders of the facial nerve was evaluated with the FDI in six studies [21,25,26,29,31,34]. All studies obtained a highly significant difference in pre- and post-intervention scores [21,25,26,29,31,34]. In three studies, the intervention with PNF obtained better results than the other group [21,25,26]. Özden et al. (2024) [34] did not obtain significant differences between groups. Tharani et al. (2022) [31] obtained better results with LLLT than ES, while Javath et al. (2021) [29] did not obtain any difference between LLLT and ES in FDI scores.

The compound muscle action potential (CMAP) was assessed in six studies [19,20,21,22,28,32]. To measure these variables, electroneurography (ENoG) [19,20,22,32] electromyography (EMG) [21,28], and a combination of both [22] were utilized.

Five studies [20,21,22,28,32] reported significant improvements, while one study did not show statistically significant results [19]. Barbara et al. [20] reported an increase in CMAP amplitude on day 15 at the end of treatment, with the CMAP amplitude ratio between the affected and healthy sides showing a 10% increase in nine patients, an 11–50% increase in eight patients, and a 50% increase in three patients. Kim and Choi [22] conducted evaluations within three weeks of PFP onset using ENoG and EMG; for those enrolled later, only EMG was used. All patients except one in the experimental group fully recovered, with improvement in facial performance across all CMAP variables and a shorter recovery time. Micarelli et al. [28] noted a significant reduction in the percentage of polyphasic potentials during voluntary activity of the orbicularis oculi and orbicularis oris muscles post-treatment. Hamed et al. [32] found that latency and degeneration percentage significantly decreased, with amplitude percentages of 74.46% for the frontalis muscle and 78.93% for the orbicularis oris muscle, both increasing by the end of treatment. Tuncay et al. [21] reported shorter distal motor latencies in the frontalis and orbicularis oris muscles, along with a significant increase in CMAP amplitudes for these muscles.

In the control group, two studies showed significant differences [21,28]. The remaining four studies did not report significant differences [19,20,22,32,34]. Tuncay et al. [21] demonstrated a significant improvement in CMAP latency of the frontalis muscle at the end of treatment, while Micarelli et al. [28] observed a significant increase in the duration of voluntary activity in the orbicularis oculi muscle.

In intergroup comparisons, Tuncay et al. [21] indicated that the CMAP amplitudes and latencies of the frontalis and orbicularis oris muscles improved significantly in the experimental group, with shorter latency times compared to the control group. Micarelli et al. [28] reported a reduction in polyphasic potentials during voluntary activity of the orbicularis oculi and orbicularis oris muscles post-treatment, along with an increase in the duration of voluntary activity in the orbicularis muscle compared to the control group.

## 4. Discussion

The purpose of this systematic review was to evaluate the efficacy of PNF and/or electrical stimulation for the treatment of PFP. Based on the results obtained, it can be stated that both physical therapy methods proved effective in both the acute and chronic phases of treatment, contributing to the recovery of muscle symmetry and mobility and reducing synkinesis. Additionally, these methods improved quality of life.

The combination of PNF with conventional therapy in the chronic phase was associated with significant improvements in facial function due to increased muscle tone, nerve regeneration, and muscle hypertrophy, which enhance symmetry and circulation activation [35]. Furthermore, physical activity from these techniques increases levels of neurotransmitters like endorphins, improving mood and reducing stress, anxiety, and depression, which directly affect quality of life [36].

In the acute phase, electrical stimulation appears to facilitate the reactivation of facial muscles, improving neuromuscular synchronization, neuroplasticity, and motor function [37,38]. This effect is attributed to the activation of glycosaminoglycans, which regulate hydration and cellular signaling, promoting tissue regeneration and reducing fibrosis and pain [39]. Consequently, reduced pain enhances the patient’s functional and emotional capacity [40].

Subcutaneous collagen injections have also proven effective for tissue regeneration and improved facial function [41]. These injections stimulate fibroblast activity and collagen synthesis, leading to better coordination and functional recovery [42]. Low-level laser treatment improved facial function and facial symmetry [29,31], coinciding with the results obtained by Amiri, P. and Fekrazad, R. in 2024 [43].

In the control group, interventions did not yield significant quality-of-life results [22,25]. This could be due to poorly defined electrical stimulation parameters, a crucial factor for effective PFP rehabilitation [44]. Additionally, in the chronic phase, intervention efficacy may be reduced due to fibrosis and muscle atrophy, underscoring the importance of early intervention [45].

Regarding synkinesis, an analysis of seven studies [22,24,25,26,27,32,33] identified significant reductions in six [24,25,26,27,32,33], attributing these results to the combined use of PNF and electrical stimulation. These techniques promote oxygenation and tissue metabolism, enhancing neuromuscular elasticity and function [46,47,48,49,50,51]. The only study that did not show a significant reduction used continuous subthreshold electrical stimulation, and the lack of reduction was potentially due to the parameters used or distal electrode placement, which may decrease the technique’s effectiveness [52,53].

In terms of movement quality, seven studies [20,21,22,23,24,25,28] reported significant improvements with PNF, PNF combined with collagen, and electrical stimulation. This can be explained by the ability of these techniques to increase muscle resistance to fatigue, facilitate the transition from fast to slow muscle fibers, and optimize motor function [54,55,56,57].

The findings of this review align with those reported by Silva et al. (2022) [58], who also concluded that PNF techniques are effective in improving muscular dysfunctions in patients with peripheral facial paralysis. However, our study expands on this by integrating combined interventions of PNF with electrical stimulation, providing additional evidence on the effectiveness of these techniques in reducing synkinesis, enhancing the quality of facial movement, and improving specific parameters such as the CMAP. Furthermore, our work includes a detailed analysis of intervention parameters (frequencies, intensities, and duration) and complementary techniques such as collagen injections, offering a broader understanding of interventions across different phases of facial paralysis. These differences highlight the contribution of this study to existing knowledge, providing new insights into integrated and personalized treatment approaches for facial paralysis.

Limitations included the use of different assessment scales, complicating result comparison. The H-B scale, although general, detects changes in facial function post-intervention [59], whereas the SFGS provides a more specific and detailed evaluation but is more complex to administer [60]. The FDI evaluates multiple subdomains, making direct comparison with other scales challenging [61,62]. Another limitation was the small sample size in several studies [20,25,26,27,28,30,34], which may have prevented statistical significance. Additionally, the lack of detailed descriptions of interventions or results in certain studies [19,21,25,26,27,30,32,33] and a follow-up period of less than a year in most were limiting factors in this review [19,20,21,22,23,24,25,26,27,28,30,32,33]. Lastly, the high heterogeneity of the studies did not allow for quantitative data analysis through meta-analysis.

Thus, there is a need for studies with larger and more representative samples, consistent electrical stimulation parameters, and standardized scales to facilitate comparison among findings. Furthermore, exploring the role of electrical stimulation in the chronic phase of PFP to determine its effectiveness in preventing long-term sequelae would be valuable.

## 5. Conclusions

Treatment with PNF and/or electrical stimulation in patients with PFP can be effective when applied early and with appropriate parameters. Low-frequency electrical stimulation should be administered daily for at least three weeks to achieve results, with a frequency between 2–4 Hz to promote trophic enhancement, circulation, and an increased pain threshold. The intensity should be perceived by the patient as comfortable, with noticeable muscle contractions, a duration of 100 ms, and an interval of 300 ms. PNF should be performed considering three fulcrums, superior, intermediate, and inferior, since most muscle fibers run diagonally, and it should be done daily for at least three weeks to begin observing changes.

PNF and/or electrical stimulation methods achieved promising results in improving quality of life, the quantity and degree of synkinesis, the quality of facial movements, and CMAP. Furthermore, combining these methods with pharmacological treatment or innovative approaches like collagen injections can further enhance quality of life and facial function by reducing disability and promoting nerve regeneration.

## Figures and Tables

**Table 1:** Risk of bias assessment using the Physiotherapy Evidence Database (PEDro) scale.

Author	1 *	2	3	4	5	6	7	8	9	10	11	Score
Cai et al. (2010) [19]	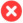	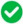	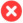	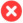	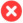	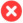	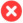	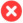	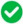	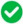	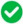	4
Barbara et al. (2010) [20]	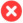	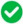	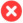	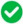	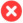	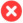	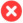	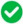	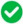	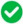	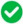	6
Tuncay et al. (2015) [21]	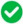	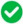	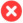	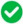	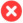	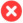	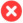	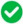	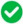	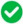	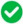	7
Kim & Choi (2015) [22]	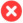	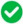	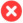	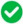	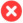	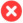	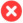	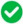	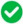	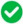	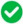	6
Monini et al. (2016) [23]	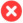	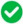	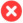	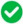	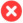	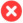	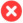	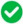	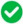	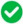	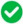	6
Monini et al. (2017) [24]	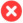	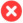	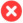	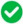	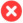	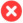	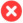	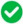	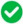	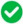	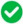	5
Ghous et al. (2018) [25]	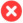	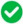	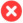	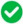	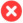	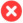	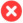	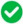	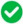	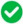	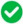	6
Khanzada et al. (2018) [26]	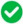	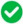	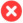	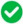	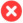	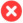	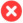	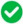	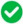	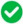	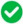	7
Marotta et al. (2020) [27]	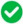	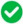	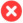	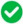	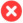	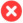	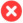	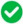	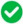	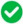	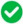	7
Micarelli et al. (2021) [28]	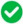	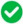	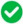	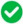	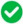	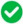	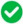	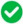	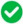	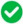	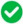	10
Javath et al. (2021) [29]	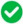	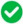	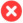	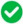	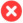	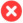	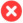	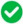	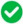	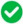	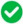	7
Avaid et al. (2022) [30]	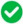	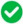	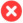	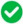	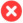	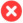	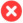	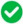	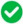	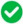	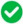	7
Tharani et al. (2022) [31]	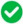	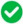	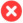	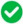	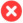	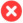	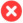	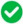	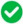	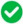	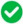	7
Hamed et al. (2023) [32]	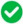	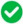	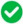	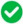	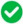	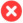	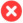	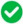	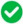	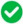	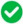	9
Alharbi et al. (2023) [33]	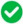	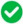	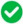	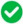	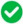	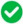	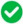	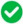	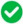	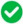	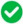	10
Özden et al. (2024) [34]	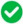	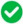	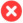	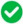	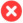	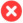	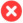	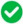	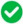	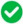	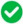	7

It shows a 1 if the item is present and a 0 if not. Items: (1) Specified selection criteria; (2) Random allocation; (3) Concealed allocation; (4) Baseline comparability; (5) Blinding of subjects; (6) Blinding of therapists; (7) Blinding of assessors; (8) Outcomes greater than 85%; (9) Intention-to-treat analysis; (10) Group comparisons; (11) Measures of data and variability. * *This item relates to external validity and is therefore not considered in the final score.*

**Table 2:** Risk of bias assessment using the RoB 2 scale.

Author	Random Sequence	Deviations from Intended Interventions	Missing Outcome Data	Measurement of the Outcome	Selection of the Reported Results	Overall
Cai et al. (2010) [19]	High	High	High	High	High	**High**
Barbara et al. (2010) [20]	High	High	High	Low	Low	**High**
Tuncay et al. (2015) [21]	Low	High	High	High	High	**High**
Kim & Choi (2015) [22]	High	High	High	High	Low	**High**
Monini et al. (2016) [23]	High	High	Low	Low	Low	**High**
Monini et al. (2017) [24]	High	High	Low	Low	Low	**High**
Ghous et al. (2018) [25]	Low	High	High	High	High	**High**
Khanzada et al. (2018) [26]	High	High	High	High	High	**High**
Marotta et al. (2020) [27]	High	High	High	High	Low	**High**
Micarelli et al. (2021) [28]	Low	Low	Low	Low	High	**Low**
Javath et al. (2021) [29]	High	Low	Low	High	High	**High**
Avaid et al. (2022) [30]	High	High	High	High	High	**High**
Tharani et al. (2022) [31]	Hight	Low	Low	Low	High	**High**
Hamed et al. (2023) [32]	Low	Low	Low	High	High	**High**
Alharbi et al. (2023) [33]	Low	Low	Low	Low	Low	**Low**
Özden et al. (2024) [34]	Low	High	Low	Low	Low	**Low**

Domains: D1, Randomization process; D2, Deviations from intended interventions; D3, Missing outcome data; D4, Outcome measurement; D5, Selection of reported results. High, high risk of bias; Uncertain, some concerns about risk of bias; Low, low risk of bias.

**Table 3:** Intervention characteristics.

Authors	Final Sample (% Women)	Intervention	Nº Sessions	Duration (Weeks)	Variables	Results
Cai et al. (2010) [19]	92 (47.8%)	EG: FT + ES + EX CG: FT + ES	ND	ND	RT, synkinesis, spasm	Functional training cannot shorten the RT, but it can reduce synkinesis and spasm.
Barbara et al. (2010) [20]	20 (50%)	EG: FT + PNF CG: FT	6	2	CMAP, HB	There were no significant differences in CMAP values. Regarding HB, the EG obtained better scores than CG.
Tuncay et al. (2015) [21]	60 (51.7%)	EG: ECR + FT + ES CG: ECR + FT	5	3	HB, FDI	HB improved in the intervention group. FDI improved significantly in both groups.
Kim & Choi (2015) [22]	60 (ND)	EG: FT + ES CG: FT	ND	ND	HB, SFGS	After 2 weeks, the EG improved significantly more in HB.
Monini et al. (2016) [23]	94 (ND)	EG: FT + PNF CG: S	ND	ND	HB, RT	EG significantly improved HB and RT scores.
Monini et al. (2017) [24]	52 (ND)	EG: FT + PNF CG: S	ND	ND	HB	EG was more likely than CG to improve in HB.
Ghous et al. (2018) [25]	20 (45%)	EG: PNF + ES CG: TP + EX + KN + ES	5	5	FDI	EG minimized FDI scores more than the CG.
Marotta et al. (2020) [27]	20 (ND)	EG: ES + EX + FT CG: EX	5	4	SB	EG obtained significantly better scores than CG on the SB.
Khanzada et al. (2018) [26]	52 (73.1%)	EG: PNF + ES CG: EX + ES	ND	3	FDI, SFGS	PNF + ES obtained better results than EX + ES in FDI and SB.
Micarelli et al. (2021) [28]	40 (42.5%)	EG: PNF + FT CG: PNF	2	8	Electrophysiological findings	The combination of PNF + FT presented better results than the use of PNF.
Javath et al. (2021) [29]	23 (ND)	EG: LLLT CG: ES	12	2	FDI, SFGS	Both groups obtained a highly significant difference in pre- and post-intervention scores. There was no difference between LLLT and ES in FDI and SB.
Avaid et al. (2022) [30]	30 (30%)	EG: PNF + ECR CG: NMR + ECR	4	4	Facial dysfunction	The combination of PNF + FT demonstrates a reduction in facial dysfunctions.
Tharani et al. (2022) [31]	30 (ND)	EG: LLLT CG: GE	3	6	FDI, SFGS	The FDI obtained a higher mean value and is more effective in EG. In SB, the EG has a higher mean value and is therefore more effective than EG.
Hamed et al. (2023) [32]	30 (53.3%)	EG: PNF + ECR CG: ECR	3	6	Electrophysiological response	% of change of latency, amplitude, and degeneration for frontalis and orbicularis oris of EG was more than CG.
Alharbi et al. (2023) [33]	62 (ND)	EG: FT + ES + NM CG: FT + ES	5	3	Facial symmetry, SFGS	GC improved facial movement and symmetry more than the CG
Özden et al. (2024) [34]	40 (45%)	EG: PNF + ECR CG: ECR	ND	4	FDI, HB, FACE, HADS, SF-12	Both groups presented improvements on all scales. There were no significant differences between groups.

EG: experimental group; CG: control group; ES: electric stimulation; S: steroids; EX: exercises; PNF: proprioceptive neuromuscular facilitation; ND: not described; NI: no intervention applies; NM: neural mobilization; NMR: neuromuscular retraining; ECR: early conventional rehabilitation; TP: tapping; KN: kinesiotaping; RT: recovery time FT: pharmacologic treatment; FDI: Facial Disability Index; FACE: Facial Clinometric Evaluation Scale; SF-12: Short Form-12; HADS: Hospital Anxiety And Depression Scale; HB: House–Brackmann; SFGS: Sunnybrook Facial Grading System; LLLT: low-level laser treatment; GE:gGalvanic electrical stimulation.

## Data Availability

The datasets used and/or analyzed during the current study are available from the corresponding author on reasonable request.

## Supplementary Materials

The following supporting information can be downloaded at https://www.mdpi.com/article/10.3390/neurolint17020017/s1: Supplementary Material S1. Search Equations.
