# Supplementary material for: Proprioceptive Neuromuscular Facilitation and/or Electrical Stimulation in Patients with Peripheral Facial Paralysis: A Systematic Review
Source: Neurol Int. 2025 Jan 23;17(2):17. doi: 10.3390/neurolint17020017 (PMC11858216; doi:10.3390/neurolint17020017)
Supplement: Supplementary file 1 [file neurolint-17-00017-s001.zip › neurolint-3367603-supplementary.pdf]

Supplementary Material 1. Search Equations.

Data base	Search Equations
**CINHAL**	*(MH “Bell’s palsy” OR MH “Facial Nerve diseases” OR MH “Cranial Nerve” OR MH “Cranial Nerve Diseases” OR MH “Facial paralysis” OR MH “Facial Nerve” OR MH “Facial Nerve Injuries”) AND (MH “Rehabilitation” OR MH “Myofunctional therapy” OR MH “Physical therapy modalities” OR MH “Electric stimulation therapy” OR MH “Exercise therapy” OR MH “Musculoskeletal Manipulations” OR MH “Exercise Movement techniques”)*
	(AB “Facial palsy” AB OR “Facial nerve palsy” OR AB “Peripheral facial paralysis”) AND (AB “Exercise” OR AB “Myofuntional exercises” OR AB “Kabat” OR AB “Electrotherapy” OR AB“Manual therapy”)
**WEB OF SCIENCE**	*TS=("Bell’s palsy” OR “Facial Nerve diseases” OR “Cranial Nerve” OR “Cranial Nerve Diseases” OR “Facial paralysis” OR “Facial Nerve” OR “Facial Nerve Injuries”) AND TS=(“Rehabilitation” OR “Myofunctional therapy” OR “Physical therapy modalities” OR “Electric stimulation therapy” OR “Exercise therapy” OR “Musculoskeletal Manipulations” OR “Exercise Movement techniques”)*
	AB=("Bell’s palsy” OR “Facial Nerve diseases” OR “Cranial Nerve” OR “Cranial Nerve Diseases” OR “Facial paralysis” OR “Facial Nerve” OR “Facial Nerve Injuries”) AND AB=(" Rehabilitation” OR “Myofunctional therapy” OR “Physical therapy modalities” OR “Electric stimulation therapy” OR “Exercise therapy” OR “Musculoskeletal Manipulations” OR “Exercise Movement techniques”)
	TS=(“Facial palsy” OR “Facial nerve palsy” OR “Peripheral facial paralysis”) AND TS=(“Exercise” OR “Myofuntional exercises” OR “Kabat” OR “Electrotherapy” OR “Manual therapy”)
	AB=(“Facial palsy” OR “Facial nerve palsy” OR “Peripheral facial paralysis”) AND AB=(“Exercise” OR “Myofuntional exercises” OR “Kabat” OR “Electrotherapy” OR “Manual therapy”)
**SPORTdiscus**	*SU “Bell’s palsy” OR SU “Facial Nerve diseases” OR SU “Cranial Nerve” OR SU “Cranial Nerve Diseases” OR SU “Facial paralysis” OR SU “Facial Nerve” OR SU “Facial Nerve Injuries”) AND (SU “Rehabilitation” OR SU “Myofunctional therapy” OR SU “Physical therapy modalities” OR SU “Electric stimulation therapy” OR SU “Exercise therapy” OR SU “Musculoskeletal Manipulations” OR SU “Exercise Movement techniques”)*
	(AB “Facial palsy” AB OR “Facial nerve palsy” OR AB “Peripheral facial paralysis”) AND (AB “Exercise” OR AB “Myofuntional exercises OR AB “Kabat” OR AB “Electrotherapy” OR AB“Manual therapy”)
**MEDLINE**	*(AB “Facial palsy” AB OR “Facial nerve palsy” OR AB “Peripheral facial paralysis”) AND (AB “Exercise” OR AB “Myofuntional exercises” OR AB “Kabat” OR AB “Electrotherapy” OR AB“Manual therapy”)*
	(MH “Bell’s palsy” OR MH “Facial Nerve diseases” OR MH “Cranial Nerve” OR MH “Cranial Nerve Diseases” OR MH “Facial paralysis” OR MH “Facial Nerve” OR MH “Facial Nerve Injuries”) AND (MH “Rehabilitation” OR MH “Myofunctional therapy” OR MH “Physical therapy modalities” OR MH “Electric stimulation therapy” OR MH “Exercise therapy” OR MH “Musculoskeletal Manipulations” OR MH “Exercise Movement techniques”)

**PUBMED**	*(Bell palsy[MeSH Terms] OR Facial Nerve Diseases[MeSH Terms] Cranial Nerve[MeSH Terms] OR Cranial Nerve Diseases[MeSH Terms] OR Facial Paralysis[MeSH Terms] OR Facial Nerve[MeSH Terms] OR Facial Nerve Injuries[MeSH Terms]) AND (Rehabilitation[MeSH Terms] OR Myofunctional therapy[MeSH Terms] OR Physical therapy modalities[MeSH Terms] OR Electric stimulation therapy[MeSH Terms] OR Exercise Therapy[MeSH Terms] OR Musculoskeletal Manipulations[MeSH Terms] OR Exercise Movement Techniques[MeSH Terms])*
	(Facial palsy[Title/Abstract] OR Facial nerve palsy[Title/Abstract] OR Peripherial facial paralysis[Title/Abstract]) AND (Exercise[Title/Abstract] OR Myofunctional Exercises[Title/Abstract] OR Kabat[Title/Abstract] OR Electrotherapy[Title/Abstract] OR Manual therapy[Title/Abstract])
**SCOPUS**	*TITLE-ABS-KEY (“Bell palsy” OR “Facial Nerve Diseases” OR “Cranial Nerve” OR “Cranial Nerve Diseases” OR “Facial paralysis” OR “Facial Nerve” OR “Facial Nerve Injuries”) AND TITLE-ABS-KEY ("Rehabilitation” OR “Myofunctional therapy” OR “Physical therapy modalities” OR “Electric stimulation therapy” OR “Exercise therapy” OR “Musculoskeletal Manipulations” OR “Exercise Movement techniques”)*
	TITLE-ABS-KEY (“Facial palsy” OR “Facial nerve palsy” OR “Peripheral facial paralysis”) AND TITLE-ABS-KEY (“Exercise” OR “Myofuntional exercises” OR “Kabat” OR “Electrotherapy” OR “Manual therapy”)
